# Genomic Sequence of the Yeast *Kluyveromyces marxianus* CCT 7735 (UFV-3), a Highly Lactose-Fermenting Yeast Isolated from the Brazilian Dairy Industry

**DOI:** 10.1128/genomeA.01136-14

**Published:** 2014-11-06

**Authors:** Wendel B. Silveira, Raphael H. S. Diniz, M. Esperanza Cerdán, María I. González-Siso, Robson de A Souza, Pedro M. P. Vidigal, Otávio J. B. Brustolini, Emille R. B. de Almeida Prata, Alexsandra C. Medeiros, Lílian C. Paiva, Moysés Nascimento, Éder G. Ferreira, Valdilene C. dos Santos, Caio R. S. Bragança, Tatiana A. R. Fernandes, Lívia T. Colombo, Flávia M. L. Passos

**Affiliations:** a Departamento de Microbiologia, Instituto de Biotecnologia Aplicada à Agropecuária (BIOAGRO), Universidade Federal de Viçosa, Viçosa, Minas Gerais, Brazil; c Núcleo de Análise de Biomoléculas (NuBioMol), Instituto de Biotecnologia Aplicada à Agropecuária (BIOAGRO), Universidade Federal de Viçosa, Viçosa, Minas Gerais, Brazil; e Departamento de Estatística, Instituto de Biotecnologia Aplicada à Agropecuária (BIOAGRO), Universidade Federal de Viçosa, Viçosa, Minas Gerais, Brazil; b Instituto de Biotecnologia Aplicada à Agropecuária (BIOAGRO), Universidade Federal de Viçosa, Viçosa, Minas Gerais, Brazil; Grupo EXPRELA, Departamento de Biología Celular e Molecular, Universidade da Coruña, Campus da Zapateira, A Coruña, Spain; d Instituto de Ciência, Engenharia e Tecnologia (ICET), Universidade Federal do Vale do Jequitinhonha e Mucuri (UFVJM), Brazil

## Abstract

Here, we present the draft genome sequence of *Kluyveromyces marxianus* CCT 7735 (UFV-3), including the eight chromosomes and the mitochondrial genomic sequences.

## GENOME ANNOUNCEMENT

Here, we present the draft genome of *Kluyveromyces marxianus* CCT 7735 (UFV-3), including the eight chromosomes and the mitochondrial genomic sequences. *Kluyveromyces marxianus* strains show both high metabolic diversity and a substantial degree of polymorphisms. This is likely to be explained by the different habitats from which it has been recovered (from plant sources and natural fermentations to ecological niches associated with warm-blooded animals, including dairy products). *K. marxianus* CCT 7735 (UFV-3), isolated from the regional Brazilian dairy industry, was selected from among other yeast isolates due to its high β-galactosidase activity, followed by a high flux of lactose assimilation and ethanol production (1). The high fermentative flux in *K. marxianus* UFV-3 seems to be favored by the high expression of key genes that encode lactose metabolism enzymes (2). It should be pointed out that opposed to other isolates and related taxa, including *Kluyveromyces lactis*, this strain is able to grow under anaerobic conditions (2). Therefore, the study of the Kluyver effect in *K. marxianus* UFV-3, i.e., the inability to transport certain sugars in the absence of oxygen, may provide new insights into carbon assimilation in yeasts of the *Kluyveromyces* genus.

On the other hand, high cell mass yields are attained when this yeast is grown in low-sugar concentrations, showing its potential as a cell factory. Besides β-galactosidase, *K. marxianus* UFV-3 exhibits high endopolygalacturonase activity. At a low growth rate, this enzyme is strongly secreted (our unpublished data).

Like the other *K. marxianus* strains, *K. marxianus* UFV-3 is capable of assimilating a variety of substrates and growing at elevated temperatures, which are desirable traits for use in biofuel production from inexpensive sources. Considering the little knowledge that exists about the genetics and physiology of *K. marxianus* UFV-3, as well as its potential to be used as a cell factory in the production of biofuel, chemicals, and recombinant proteins, we sequenced its genome in order to gain insights about its biochemical and genetic traits.

Three libraries with insert sizes of 500 nucleotides (nt), 2 kb, and 5 kb were constructed, amplified, and sequenced in Illumina HiSeq 2000 by Sistemas Genómicos (Valencia, Spain), producing sequence data containing 51 million paired reads with 100 nt. To assembly the chromosomes and mitochondrial genomic sequences of *K. marxianus* UFV-3, the sequences of *K. marxianus* DMKU3-1042 (Genbank accession no. AP012213 to AP012221) were selected as references. The assembly was performed using a variant calling approach. The reads were mapped in the references using CLC Genomics Workbench version 6.5.1 (CLC bio), and the contigs were reconstructed using Bcfutils of SAMtools version 1.4 (3). This assembly produced nine contigs with overall G+C contents of 39% for chromosomes and 13.5% for the mitochondrial sequences and mean coverages of 385-fold for the chromosomes and 841-fold for the mitochondrial sequences. The contigs were analyzed using Augustus version 2.5.5 (4, 5) to predict the genes with *K. lactis* trained data. The proteins encoded by the 4,787 predicted genes were functionally annotated using BLAST searches (http://blast.ncbi.nlm.nih.gov/) and HMMER (6). Most of the predicted proteins (~97%) presented high similarity to *K. marxianus* DMKU3-1042 proteins, and some (~3%) were similar to *K. lactis* NRRL Y-1140.

### Nucleotide sequence accession numbers.

The complete genomic sequences of the *K. marxianus* CCT 7735 (UFV-3) chromosomes and mitochondrial genome have been deposited in GenBank under the accession numbers CP009303 to CP009311.
